# ASO Author Reflections: Chyle Leak: No Long-Term Impact on Survival

**DOI:** 10.1245/s10434-020-09410-9

**Published:** 2020-11-21

**Authors:** Pamela Milito, Alexander W. Phillips

**Affiliations:** Northern Oesophago-Gastric Cancer Unit, Royal Victoria Infirmary, Queen Victoria Road, Newcastle-upon-Tyne, UK

## Past

Chyle leak is a well-recognised complication of transthoracic esophagectomy with a reported incidence of 1 to 9%.^1^ This complication has been associated with a higher rate of morbidity and mortality.^2^ A variety of strategies have been postulated in the past, which have included reoperation, embolization, and dietary modulation with use of total parenteral nutrition or enteral feeding using medium-chain triglyceride (MCT) diets.^3^^,^^4^ However, treatment of this complication needs to be tailored to the individual clinical scenario to determine which strategy may be most appropriate.

## Present

The present study indicates that surgical outcomes of patients developing chyle leak were similar to those who had an uneventful surgery, except for the length of critical care stay which was longer for those developing a chyle leak.^5^ More importantly, a chyle leak was not associated with any detrimental long-term effect. In particular, there was no significant difference in overall survival regardless of tumour histology between patients who developed a leak and those who did not. Conservative treatment, with the implementation of MCT feed, may address many leaks that are encountered. However, high-volume leaks are likely to require surgical intervention, which should not be feared, because it is safe and effective.

## Future

An early detection and standardized treatments for chyle leakage after transthoracic esophagectomy may help to improve the morbidity and mortality of this complication. The range of available approaches vary. However, minimally invasive treatment, such as embolization of the thoracic duct, is not widely available. In the future, newer surgical techniques, such as robotic surgery or the use of augmented reality, may help to provide a better intraoperative identification of problems that will reduce the incidence.
